# Objectification decreases prosociality: the mediating role of relative deprivation

**DOI:** 10.3389/fpsyg.2023.1120513

**Published:** 2023-06-05

**Authors:** Zaixuan Zhang, Zhansheng Chen, Kai-Tak Poon, Tonglin Jiang

**Affiliations:** ^1^Department of Psychology, The University of Hong Kong, Pokfulam, Hong Kong SAR, China; ^2^Department of Psychology and Centre for Psychosocial Health, The Education University of Hong Kong, Tai Po, Hong Kong SAR, China; ^3^Beijing Key Laboratory of Behavior and Mental Health, School of Psychological and Cognitive Sciences, Peking University, Beijing, China

**Keywords:** interpersonal relation, objectification, relative deprivation, prosocial intention, prosocial behavior

## Abstract

Objectification denies individuals’ personhood and renders them as tools for facilitating others’ goal achievement. With two studies (*N* = 446), the present investigation aimed to contribute to the literature by testing whether and how objectification impacts prosociality, including prosocial intention and prosocial behavior. Study 1, with a correlational design, aimed to test whether participants with greater experience of objectification would report lower levels of prosociality, and to test whether participants’ relative deprivation could account for the proposed association between objectification and prosociality. To further test these associations and provide causal evidence, in Study 2, we manipulated objectification by asking participants to imagine future objectification experiences. These studies converged in support of the negative relationship between objectification and prosocial intention, as well as the mediating role of relative deprivation. Regarding prosocial behavior, our findings support a mediating mechanism between objectification and prosocial behavior, although the evidence for the effect of objectification on prosocial behavior is not sufficient. These findings enrich our understanding of the consequences of objectification, while highlighting interpersonal processes’ contribution to prosocial intention and behavior. The limitations and potential future directions were discussed.

## Introduction

When people are objectified, they are treated merely as things or tools that can aid others’ goal achievement, and their autonomy, needs, feelings, and opportunities are neglected (Nussbaum, 1995; Fredrickson and Roberts, 1997). Recent research has shown that, aside from affecting intrapersonal processes, objectification can also impair people’s interpersonal relations by increasing external aggression (Poon et al., 2020). The present research aimed to further investigate the impact of objectification on interpersonal processes. In particular, we focused on whether and how objectification influences prosociality, which plays an important role in promoting positive interpersonal relations (Estrada-Hollenbeck and Heatherton, 1998). Previously, different group of researchers have revealed individuals’ declined prosociality after experiencing maltreatments, such as social exclusion (e.g., Twenge et al., 2007), peer bullying (e.g., Raskauskas et al., 2010), and racial discrimination (e.g., Davis et al., 2016). Therefore, we believe objectification, as another experience of maltreatments, can also decrease individuals’ prosociality.

### Objectification and its negative consequences

The experience of being objectified is a common one. For instance, employees can be treated as mere instruments to aid the financial success of their employers, students can be treated by their classmates as note-takers, and so on. Originating from women’s studies, a large body of literature has shown the negative effects of being viewed as a sexual object (e.g., Moradi and Huang, 2008; Calogero et al., 2009). For instance, sexual objectification can lead to sinful feelings among those victimized women (Chen et al., 2013). Moreover, sexual objectification also reduces women’s performance on cognitive tasks (Fredrickson et al., 1998; Quinn et al., 2006) and leads them to perceive themselves as less likable and to avoid interacting with men (Teng et al., 2015).

However, the consequences of non-sexual objectification are less well-known, despite objectification’s prevalence (Holland et al., 2017). Some scholars found that engaging in an objectifying activity would increase individuals’ conforming behavior, and their self-objectification after the objectifying task could account for this effect (Andrighetto et al., 2018). More recently, it has also been shown that objectification reduces people’s sense of authenticity, which further decreases their subjective well-being (Cheng et al., 2022). Moreover, another group of researchers revealed that objectification could trigger aggression: because it impairs people’s sense of control, and objectification made individuals become more aggressive to restore their control (Poon et al., 2020).

Relative deprivation was defined as a judgment that one or one’s ingroup is in a disadvantageous position (Smith and Pettigrew, 2015). According to Smith and Pettigrew (2015), relative deprivation contains three segments in sequence. First, individuals’ cognitive comparison would be elicited by their particular experiences. Secondly, through cognitive appraisals during the comparison process, individuals would realize their disadvantaged conditions. Finally, individuals’ justice-related emotions would be aroused. Although scholars mainly focus on the consequences of relative deprivation (e.g., addiction to alcohol and drug; Baron, 2004), some researchers have addressed its antecedents. For example, Shedd (2008) indicated that the post–Soviet Union political reality induce a relative deprivation among people in Chechen. Additionally, it has been detected that participants who reported lower subjective socioeconomic status suffer from a higher level of relative deprivation (Callan et al., 2015). Recently, Jiang and Chen (2020) found that ostracism experience could also make individuals feel relatively deprived. Given that, it seems individuals suffering from negative life events are more likely to hold stronger relative deprivation.

Because objectification involves the attention to one’s usefulness in facilitating goal achievement as well as the denial of personhood, its victims’ autonomy, needs, feelings, and opportunities are neglected. Objectification makes its victims tools for the purposes of its conductors, and its extreme form is slavery (Nussbaum, 1995). Indeed, objectification can result in a decreased sense of control (i.e., control deprivation) because the social interactions of objectified individuals are all manipulated and controlled by the objectifiers (Poon et al., 2020). Thus, objectified individuals would feel that they were treated unjustly, and that their autonomy was deprived. As argued by Baldissarri et al. (2021), treating others as fungible tools involves a pervasive dehumanizing perception. Empirically, the authors also found that more objectified targets were perceived as more aliens with lower agency and lower experience than their counterparts (Baldissarri et al., 2017). As such, objectified individuals felt that their feelings and needs, which made them whole human beings, were all ignored. It is thus reasonable to expect that objectified individuals would perceive themselves as disadvantaged relative to others, and that they would experience a sense of relative deprivation. Therefore, we hypothesized that people would feel a sense of relative deprivation after being objectified.

### Objectification, relative deprivation, and prosociality

The sense of relative deprivation triggered by objectification may further influence prosociality, which refers to the expression of behaviors (e.g., donating, comforting, and helping) that benefit other people (e.g., Penner et al., 2005; Caprara et al., 2012; Galen, 2012). According to evolutionary theory, the prosocial act is an important factor in human beings’ evolutionary success (e.g., Barrett et al., 2002). As our ancestors lived in clans with almost only their relatives, only those clans whose members offered help to each other could survive (Penner et al., 2005). Prosocial behaviors may also contribute to one’s own survival individually by helping increase individuals’ ingroup reputations (Wedekind and Braithwaite, 2002). From an intergroup perspective, groups with an altruistic atmosphere would be more powerful and gain more benefits during intergroup competition than their selfish counterparts (Sober and Wilson, 1999). As such, helping can even be considered a kind of instinct of humanity. Nevertheless, some other external elements could influence individuals’ prosociality.

Reciprocity, especially positive reciprocity, is another factor that is believed to drive prosociality and help to maintain cooperative systems in our society (Fehr and Fischbacher, 2003). Positive reciprocity refers to a series of responses to others’ kindness; those behaviors or intentions are also known as reciprocal altruism (Falk and Fischbacher, 2006). Over the course of development of human society, reciprocity has emerged as a kind of social norm (Perugini et al., 2003), which means that kindness is generally rewarded with kindness. It has been found that people are more willing to be helpful when encountering those who have helped others (Boster et al., 2001). However, when it comes to individuals with objectification experiences, the reciprocity norm may no longer work. As aforementioned, objectified individuals are more likely to suffer from relative deprivation, and such feelings make them focus more on themselves but less on others. For example, relative deprivation has been found to make individuals prioritize self-interest over others (Zhang et al., 2016), and other scholars detected a significant negative association between relative deprivation and a zero-sum mindset (Dong et al., 2023). In response, with more self-orientation and less other-orientation, the reciprocity norm is broken. And objectified individuals become less prosocial, to protect their own interests from further possible injury. Additionally, according to Relative Deprivation Theory (Smith et al., 2012), suffering from relative deprivation means arousal in justice-related emotions, such as frustration, dissatisfaction, and disappointment. On that occasion, individuals would hold the opinion that they do not deserve such an experience. Therefore, in order to achieve justice and fairness, they may employ more individually oriented behaviors. For instance, adults who felt relatively deprived were more likely to conduct ate rape (Boeringer, 1992), and children who suffered from relative deprivation conducted more bullying (Breivik and Olweus, 2006). Given that, as individuals are treated as a target with unfair status (i.e., treated as instruments), individuals’ prosociality would drop after objectification.

Moreover, the negative association between relative deprivation and prosociality was repeatedly reported in the literature. Zhang et al. (2016) found that both self-reported and manipulated relative deprivation could result in lower prosociality, and they argued that the reduced willingness to sacrifice one’s own interests after relative deprivation and its central emotional components (i.e., anger and resentment) would account for their findings. Others replicated this effect and further proposed subjective socioeconomic status (SES) as a moderator (Callan et al., 2017). Recently, with an adolescent sample, Xiong et al. (2021) replicated the relation between relative deprivation and decreased prosocial tendency, and they further found that perceived social support could help interpret the association between relative deprivation and prosocial tendencies.

Given the hypothesized effect of objectification on relative deprivation as well as the strong negative association between relative deprivation and prosociality, we further hypothesized that non-sexual objectification experiences would decrease individuals’ prosociality, and that relative deprivation would account for the effect of objectification on prosociality. That is, objectification triggers a sense of relative deprivation, which further diminishes prosociality.

### The current research

In the current research, we aimed to investigate whether and how non-sexual objectification influences prosociality. Firstly, using a correlational design, we tested whether participants who reported higher levels of objectification experience would report lower levels of prosociality, including prosocial intention and prosocial behaviors. Additionally, we further examined whether participants’ perceived relative deprivation could account for the association between objectification and prosociality (Study 1). Then, to further replicate and provide causal evidence for the relation we detected in Study 1, we manipulated participants’ objectification experience with an imagining task (i.e., participants were asked to imagine future objectification experiences) and tested their prosocial intention and prosocial behavior with different measurements (Study 2).

## Study 1

In this study, we initially explored the relationship between objectification experience and prosociality with a correlational design. We predicted that suffering from higher levels of objectification would result in lower levels of prosociality, including prosocial intention and behavior. Further, we tested whether relative deprivation could account for the association between objectification and prosociality.

### Participants

In total, 270 American participants were recruited using Amazon’s Mechanical Turk (Rand, 2012), and they participated in exchange for 0.3 USD, while 19 were excluded for failing the attention check. Among the participants, 68.9% were White, 6.8% were Black, 12.7% were Asian, 5.2% were Latin, 3.6% were of other ethnicities, and another 2.8% preferred not to answer. A sensitivity test (Faul et al., 2007) showed that our final sample size of 251 (148 women, *M*_age_ = 43.1, *SD*_age_ = 13.2, *M*_SES_ = 5.69, *SD*_SES_ = 1.93) could provide 80% power to detect an effect of *ρ* = 0.15 (small-medium; *α* = 0.05).

### Procedures and measurements

Generally, after the consent forms, participants were exposed to different scales separately. First, we assessed participants’ experience of objectification was assessed; thereafter, their relative deprivation was measured. Finally, they reported their prosocial intention and recent prosocial behavior, along with their demographic information (i.e., gender, race, age, and subjective socioeconomic status). To assess participants’ subjective socioeconomic status, a previous measurement was employed (Adler et al., 2000). Specifically, participants were presented with a figure of a 10-level ladder, which represents the social hierarchy in society. Participants were asked to indicate the rung that best represents where they are in society.

#### Objectification experience (*M* = 4.14, *SD* = 0.97, *α* = 0.82)

In response to ten items adapted from a measurement developed in past research (Gruenfeld et al., 2008; e.g., “Other people tend to contact me only when they need something from me.”), participants expressed the extent to which they had experienced objectification, answering on a scale ranging from *strongly disagree* (i.e., “1”) to *strongly agree* (i.e., “7”) for each item.

#### Relative deprivation (*M* = 3.41, *SD* = 1.27, *α* = 0.76)

We applied the five-item Personal Relative Deprivation Scale originated by Callan et al. (2017; e.g., “I feel deprived when I think about what I have compared to what other people like me have.”). Participants reported their feelings of relative deprivation, by responding on a scale ranging from *strongly disagree* (i.e., “1”) to *strongly agree* (i.e., “7”) for each item.

#### Prosocial intention (*M* = 5.41, *SD* = 1.01, *α* = 0.84)

Seven items were adapted from the Prosocial Behavioral Intentions Scale (Baumsteiger and Siegel, 2018; e.g., “Help care for a sick friend or relative.”). Participants expressed their willingness to engage in each prosocial behavior, responding on a scale ranging from *strongly disagree* (i.e., “1”) to *strongly agree* (i.e., “7”) for each item.

#### Recalled prosocial behavior (*M* = 4.51, *SD* = 1.26, *α* = 0.71)

The five-item Prosocial Index (Charities Aid Foundation, 2019) was applied (e.g., “Donated money to a charity.”). Participants indicated how often they engaged in particular prosocial behaviors in the last 3 months, by responding on a scale ranging from *not at all* (i.e., “1”) to *very often* (i.e., “7”) for each item.

### Results and discussion

Overall, using all demographic variables (i.e., gender, age, race, and subjective socioeconomic status) as covariates, we conducted several analyses with multiple linear regression. It was revealed that participants’ objectification experience negatively associated with their prosocial intention, *b* = −0.207, *t* = −3.24, *p* = 0.001, 95% CI [−0.346, −0.084], *R*^2^ = 0.039, but not their recalled prosocial behavior, *b* = −0.042, *t* = −0.64, *p* = 0.525, 95% CI [−0.221, 0.113], *R*^2^ = 0.012. Consistent with our hypothesis regarding the association between objectification and prosociality, participants with higher levels of objectification reported lower levels of prosocial intention. However, participants’ objectification experience did not correlate with their recalled prosocial behaviors.

Further analyses, after all the demographic variables were included as covariates, revealed that participants’ objectification experience positively associated with their sense of relative deprivation, *b* = 0.234, *t* = 3.74, *p* < 0.001, 95% CI [0.144, 0.465], *R*^2^ = 0.084. Participants with higher levels of objectification experience reported a greater sense of relative deprivation. In addition, there was a significantly negative association between participants’ relative deprivation and their prosocial intention, *b* = −0.249, *t* = −3.95, *p* < 0.001, 95% CI [−0.297, −0.099], *R*^2^ = 0.058, as well as another negative relation between their relative deprivation and recalled previous prosocial behavior, *b* = −0.237, *t* = −3.73, *p* < 0.001, 95% CI [−0.359, −0.111], *R*^2^ = 0.040. This meant that individuals with a higher magnitude of relative deprivation tended to carry lower prosocial intention and had conducted fewer prosocial behaviors in the past (see Table 1 for the correlation matrix).

**Table 1:** Correlations matrix of study 1 (*N* = 251).

No.	Variables	*α*	*M* (*SD*)	1	2	3	4	5	6	7	8
1	Objectification experience	0.82	4.14 (0.79)	—							
2	Relative deprivation	0.76	3.41 (1.27)	0.261^***^	—						
3	Prosocial intention	0.84	5.79 (1.01)	−0.225^**^	−0.256^***^	—					
4	Recalled prosocial behavior	0.71	4.51 (1.26)	−0.053	−0.243^***^	0.451^***^	—				
5	Gender	/	/	−0.087	−0.038	0.061	0.022	——			
6	Race	/	/	0.108	−0.047	−0.061	0.023	0.127^*^	—		
7	Age	/	43.06 (13.21)	−0.225^***^	−0.210^***^	0.099	0.068	0.110	0.110	—	
8	Subjective-SES	/	5.69 (1.93)	−0.008	−0.048	0.045	0.028	0.070	0.113	−0.012	—

SES = subjective socioeconomic status. ^*^p < 0.05; ^**^p < 0.01; and ^***^p < 0.001.

**Figure 1:**
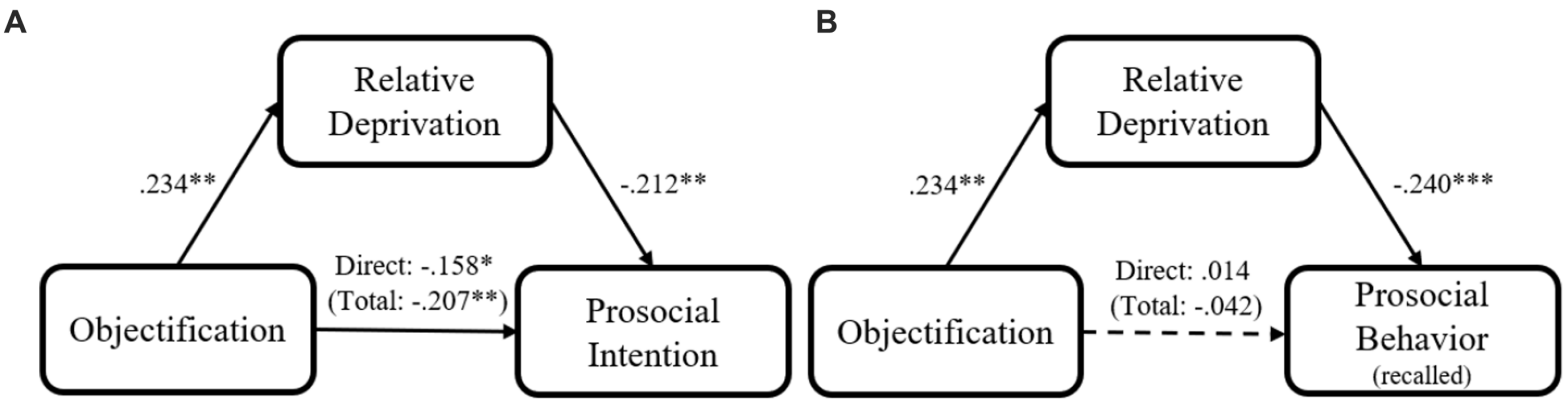
Mediation models of participants’ objectification experience on their prosocial intention **(A)** Recalled prosocial behavior **(B)** for Study 1 (Coefficients are standardized; ^*^p < 0.05; ^**^p < 0.01; and ^***^p < 0.001).

Next, we examined the potential mediating role of participants’ relative deprivation in the relation between their objectification experiences and their prosociality. Several mediation analyses using PROCESS Model 4 (Hayes, 2012; 5,000 iterations, bias corrected) were conducted, with all demographic variables included as covariates. We found that relative deprivation could partially mediate the association between participants’ objectification experience and their prosocial intention, indirect effect = −0.050, *SE* = 0.024, 95% CI = [−0.099, −0.004] (standardized; Figure 1A). Moreover, the relation between participants’ objectification experience and their recalled prosocial behavior could be fully accounted for by their relative deprivation, indirect effect = −0.056, *SE* = 0.033, 95% CI = [−0.137, −0.008] (standardized; Figure 1B).

The present study provided initial evidence for the relationship between one’s objectification experience and prosociality. In line with our hypothesis, objectification experience was negatively associated with prosocial intention. We also detected two models of mediation, in which relative deprivation mediated the relation between objectification experience and prosocial intention (partially mediation) and that between objectification experience and recalled prosocial behaviors (fully mediation).

It is interesting to note that the association between objectification and past prosocial behavior was not significant, although the mediating role of relative deprivation in their association was significant. This finding suggested that the effect of objectification on prosocial behavior might be underpowered, among several other potential reasons, and that increasing the sample size may lead to a significant association between objectification and prosocial behavior. However, it is also possible that the current reported mediation role of relative deprivation on prosocial behavior could be a random error. In addition, the measurement issue could be another possible reason. Prosocial behavior could symbolize individuals’ morality, and maintaining one’s moral image has been argued to be a basic need of human beings (Prentice et al., 2019). As such, participants may over-calming the “good things” they had done when recalling past prosocial behaviors. Nevertheless, our results indicate a mediating mechanism at work between objectification and prosociality, including prosocial intention and prosocial behavior, although there is not enough evidence to support an association between objectification and prosocial behavior.

In the next study, we manipulated objectification to further test our hypotheses with causal evidence. Besides, we applied new assessments of prosocial intention and prosocial behavior. Additionally, regarding the increased negative emotion after objectification experience (Poon et al., 2020; Study 1) and the negative effect of negative emotion on prosociality (e.g., Bagozzi and Moore, 1994; Malti et al., 2009), there is a possibility that negative emotion could also mediate the relationship between objectification and prosociality. Therefore, in the next study, following the practice of Poon et al. (2020), we included negative emotions as an alternative mediator.

## Study 2

In this study, we aimed to manipulate objectification with an imagining task (Poon et al., 2020, Experiment 2). In that paradigm, participants in the objectification condition imagined an identical scenario, which could elicit a similar magnitude of objectification feelings among them. Another more recently developed measurement (Jiang and Sedikides, 2021) was employed to detect participants’ prosocial intentions. We predicted that those who imagined being objectified by others (vs. imagined an experience of being treated decently) would show lower prosocial intention and fewer prosocial behaviors, while relative deprivation may continue to serve as a mediator in such a process.

### Participants

In total, 200 American participants were recruited using Amazon’s Mechanical Turk (Rand, 2012), and they participated in exchange for 0.3 USD, while five were excluded because of failing the attention check. Among the participants, 64.1% were White, 8.7% were Black, 9.7% were Asian, 6.7% were Latin, 9.2% were of other ethnicities, and another 1.5% preferred not to answer. A sensitivity test (Faul et al., 2007) showed that our final sample size of 195 (119 women, *M*_age_ = 40.7, *SD*_age_ = 14.1, *M*_SES_ = 5.50, *SD*_SES_ = 1.97) could provide 80% power to detect an effect of *η*_p_^2^ = 0.039 (small-medium; *α* = 0.05).

### Procedures and materials

At the beginning of the experiment, participants were told that they were going to take part in an imagining task to test their imagination. After the consent form, participants were randomly assigned to two different conditions. In the objectification condition, participants were asked to imagine experiences of objectification in their college (i.e., in which they were treated as a tool by their classmates) and at their internship company (i.e., in which they were treated as a tool by their intern supervisor; see supplemental material for details). Meanwhile, their counterparts in the non-objectification condition were asked to imagine a similar experience in their college and internship company, but in which they were treated decently. After that, all participants responded to three manipulation check items (e.g., “I feel like I am being treated as an object”; *M* = 4.53, *SD* = 1.83, *α* = 0.85).

Then, all participants were asked to report their relative deprivation (*M* = 3.34, *SD* = 1.40, *α* = 0.85), while the measurement was the same as the one used in Study 1 (Callan et al., 2017; e.g., “I feel deprived when I think about what I have compared to what other people like me have”; *1 = strongly disagree, 7 = strongly agree*). Afterward, their negative emotions (*M* = 3.85, *SD* = 0.49, *α* = 0.94) were measured to serve as another covariate (e.g., “I feel sad”; *1 = strongly disagree, 7 = strongly agree*). Next, they were exposed to 14 items to test their prosocial intention (*M* = 8.26, *SD* = 1.83, *α* = 0.95) developed by Jiang and Sedikides (2021; e.g., “I feel I would do what I can to help others avoid getting into trouble”; *1 = not at all, 11 = very much so*). Then, participants’ prosocial behavior was tested with a donation scenario, in which participants reported the amount of participation fee (0USD–0.3USD) they would like to donate to a charity (the amount of donation was standardized). Finally, participants’ demographic information was collected (same as Study 1), and then they were thanked and debriefed (their participation fee was paid as initially agreed).

### Results and discussion

#### Manipulation checks

Participants in the objectification condition (*M* = 6.06, *SD* = 0.99) reported significantly greater objectification feeling than their counterparts in the non-objectification condition did (*M* = 2.85, *SD* = 0.72), *t* (193) = 25.73, *p* < 0.001, *d* = 3.69. This result indicates that our manipulation was effective.

#### Relative deprivation

Participants in the objectification condition (*M* = 3.62, *SD* = 1.43) reported significantly higher relative deprivation than their counterparts in the non-objectification condition did (*M* = 3.04, *SD* = 1.31), with all demographic variables (i.e., gender, age, race, and subjective socioeconomic status) included as covariates in the analysis, *F* (1, 189) = 8.20, *p* = 0.005, *η*_p_^2^ = 0.0.42. However, such an effect could be qualified by participants’ age, *F* (1, 189) = 13.82, *p* < 0.001, *η*_p_^2^ = 0.0.68, but not other demographics, *F_s_* (1, 189) < 3.39, *p_s_* > 0.067. It suggested that elders were less likely to feel relatively deprived.

#### Negative emotion

Participants in the objectification condition (*M* = 3.91, *SD* = 0.54) reported negative emotions similar to their counterparts in the non-objectification condition (*M* = 3.79, *SD* = 0.44), with all demographic variables included as covariates in the analysis, *F* (1, 189) = 2.05, *p* = 0.154, *η*_p_^2^ = 0.011.

#### Prosocial intention

Participants in the objectification condition (*M* = 8.02, *SD* = 1.66) reported less prosocial intention than their counterparts in the non-objectification condition (*M* = 8.52, *SD* = 1.97), after all demographic variables and negative emotion were included as covariates in the analysis, *F* (1, 188) = 4.28, *p* = 0.040, *η*_p_^2^ = 0.029. Besides, such an effect could be qualified by participants’ gender (*F* (1, 188) = 5.71, *p* = 0.018, *η*_p_^2^ = 0.022) and age (*F* (1, 188) = 6.83, *p* = 0.010, *η*_p_^2^ = 0.035), but not their race (*F* (1, 188) = 0.44, *p* = 0.509, *η*_p_^2^ = 0.002) and SES (*F* (1, 188) = 0.18, *p* = 0.672, *η*_p_^2^ = 0.001). It means that females and elders were more likely to express prosocial intentions than their counterparts.

#### Prosocial behavior

Participants in the objectification condition (*M* = 0.008, *SD* = 1.05) reported prosocial behavior similar to their counterparts in the non-objectification condition (*M* = −0.009, *SD* = 0.943), after all demographic variables and negative emotion were included as covariates in the analysis, *F* (1, 188) = 0.001, *p* = 0.973, *η*_p_^2^ = 0.000. Besides, such an effect could not be qualified by participants’ demographics, *F_s_* (1, 188) < 3.27, *p_s_* > 0.076. Additionally, there was a significant association between participants’ prosocial intention and prosocial behavior, *r* = 0.213, *p* = 0.003.

Additionally, we detected a significant association between participants’ relative deprivation and their prosocial intentions, *b* = −0.372, *t* = −3.78, *p* < 0.001, 95% CI [−0.426, −0.144], *R*^2^ = 0.130, as well as another significant association between participants’ relative deprivation and their prosocial behaviors, *b* = −0.149, *t* = −2.84, *p* = 0.005, 95% CI [−0.353, −0.064], *R*^2^ = 0.084.

**Figure 2:**
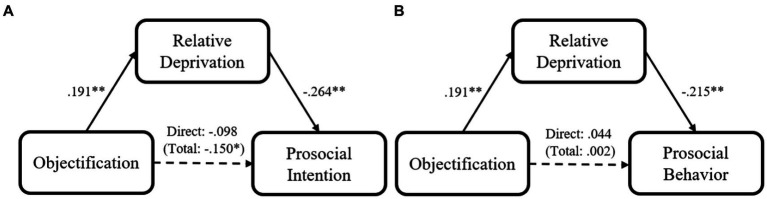
Mediation models of participants’ objectification experience on their prosocial intention **(A)**/Prosocial behavior **(B)** for Study 2 (Neutral condition = 0, Objectification condition = 1; coefficients are standardized; ^*^p < 0.05; ^**^p < 0.01; and ^***^p < 0.001).

We then tested whether relative deprivation mediated the effect of objectification on prosocial intention. Using PROCESS Model 4 (Hayes, 2012; 5,000 iterations, bias corrected) we conducted mediation analysis (with all the demographic variables included as covariates), which indicated that the experience of objectification could decrease participants’ prosocial intention *via* relative deprivation, indirect effect = −0.051, *SE* = 0.082, 95% CI = [−0.349, −0.027] (Figure 2A). Further, we conducted another mediation analysis with the same covariates, again using PROCESS Model 4 (Hayes, 2012; 5,000 iterations, bias corrected), to test whether relative deprivation mediated the effect of objectification on prosocial behavior with another mediation analysis with the same covariates using PROCESS Model 4 (Hayes, 2012; 5,000 iterations, bias corrected). Even though the main effect of objectification on prosocial behavior was not significant, relative deprivation had a significant fully mediating effect, indirect effect = −0.041, *SE* = 0.044, 95% CI [−0.169, −0.005] (Figure 2B). In addition, another two mediation analyses indicated that negative emotion could not account for the relationship between objectification and prosocial intention indirect effect = 0.062, *SE* = 0.050, 95% CI = [−0.036, 0.161], or prosocial behavior, indirect effect = −0.010, *SE* = 0.016, 95% CI = [−0.041, 0.022].

Together, the study further supported our hypotheses with causal evidence. Objectified participants, relative to their neutral counterparts, reported lower levels of prosociality, and such an effect was explained by participants’ higher levels of relative deprivation after objectification. It should be pointed out that, as in Study 1, there was not enough evidence to support the main effect of objectification on prosocial behavior.

## General discussion

In the current research, we provided some evidence that objectification can decrease individuals’ prosocial intentions, which may harm their interpersonal relationships. Specifically, in Study 1, those who reported higher levels of objectification experiences reported lower levels of prosocial intention. It was also found that relative deprivation accounted for the association between objectification and prosocial intention. In addition, we identified the mediating mechanism of relative deprivation in the relationship of objectification and prosocial behavior. Further, using an experimental design, Study 2 replicated the negative influence of objectification on prosocial intention. Those who imagined an objectification experience, relative to those who imagined a non-objectification experience, reported lower levels of prosocial intention, which was accounted for by relative deprivation. Regarding prosocial behavior, although there was not enough evidence to support the effect of objectification on prosocial behavior, we found relative deprivation to have a consistently mediating role.

### Implications of the present research

Our current research can contribute to the literature in several ways. First, our findings enrich the literature on objectification by further detailing its negative impact on interpersonal processes. Prior to our research, it was reported that objectification could result in aggression toward others (Poon et al., 2020). While in the current research, the negative effect of objectification on prosociality provides another perspective, in which individuals’ interpersonal relations could also be impaired passively. As prosociality plays an important part in the cooperation of human society (Simpson and Willer, 2015), non-prosocial individuals are less likely to be regarded as valued by others, resulting in fewer and worse potential interpersonal interactions.

Secondly, the current research contributes to the literature on objectification by providing new insights into its negative impacts. Together with the work of Poon et al. (2020) and Cheng et al. (2022), the current research suggests that objectification may have a profound impact on interpersonal processes. Moreover, on a methodological level (and in line with these two groups of scholars) the previously used manipulation of objectification (i.e., the imagining paradigm) was also effective in the current research (*η*_p_^2^ = 0.774), which could be further applied in following research on objectification.

In addition, we proposed relative deprivation as an interpretation for the negative relation between objectification and prosociality, and detected its mediating role in both studies. In line with previous scholars (e.g., Nussbaum, 1995), these findings imply that objectification is indeed a process that includes exploitation and oppression, which allows its conductors to take advantage of its victims. The findings may also help interpret the negative association between post-objectification authenticity and well-being proposed by Cheng et al. (2022). Specifically, as people expect to be treated fairly in their daily lives (Penner et al., 1997), after their authenticity decreases through objectification, they will feel relatively deprived, which leads to a decline in their well-being. In contrast, consistent with a previous finding (Poon et al., 2020; Experiment 2), we found negative emotions could not be significantly influenced by objectification, nor could they serve as a significant mediator in the relation between objectification and reduced prosociality. Although relative deprivation is a concept that overlaps the emotional and cognitive domains (Smith et al., 2012), the insignificant role of negative emotions suggests that some subtler feelings (e.g., the feeling of being unfairly treated, the feeling of being dehumanized) that could be captured by relative deprivation may account more for individuals’ reactions to objectification than general negative moods.

Finally, regarding the consistent relation between objectification and prosociality, our current studies suggest another potential path to promote prosociality. First, prosociality could be facilitated by reducing objectification. For example, as the experience of being grateful could make individuals less likely to objectify others (Shi et al., 2022), people could be educated to integrate more habitual gratitude into their daily lives. Besides, individuals should be alert and avoid others’ potential objectification towards them. Additionally, on a more macroscopic level, every entity in society should adopt a focus on others’ psychological features and internal feelings rather than their usefulness (Briñol et al., 2017), which could reduce objectification and promote a prosocial atmosphere.

### Limitations and future directions

Despite the consistent results found by our research, it is not without limitations that present opportunities for improvement. First, although the mediating role of relative deprivation in the relationship between objectification and lower prosociality was repeatedly detected by different studies, it should still be interpreted with caution. There could also be other internal processes with potential. For example, Cheng et al. (2022) indicated that objectification would reduce individuals’ perceived authenticity, while an authentic self was found to be related to higher general prosociality (Jiang and Sedikides, 2021). Therefore, an alternative explanation of the decrease in prosociality caused by the objectification experience might point to individuals’ declined authenticity as a cause. Future research may explore other potential internal processes and manipulate relative deprivation and other potential mediators to discern a more convincing causal link. Additionally, as the effect of negative emotions could not independently explain the negative relation between objectification and prosociality, more attention should be paid to the cognitive aspect of relative deprivation. Specifically, future research may investigate whether the perceived reciprocity reduced by objectification could do a negative impact on prosociality alone, or whether this altered perception works together with negative emotion *via* relative deprivation. Alternatively, regarding the internalization of objectification (e.g., Fredrickson and Roberts, 1997), and the negative impact of self-objectification on one’s emotions (Roberts and Gettman, 2004), it would be reasonable to assume that self-objectification may also partially account for the relation between the experience of objectification and lower prosociality, a link that could be explored in the future. Even so, regarding the steady relation between higher relative deprivation and lower prosociality (e.g., Callan et al., 2017; Xiong et al., 2021), we believe that relative deprivation is still a plausible mediator.

Secondly, even after taking negative moods into account, we have not ruled out other factors induced by the experience of objectification, which may also negatively impact individuals’ prosociality. Recently, Dvir et al. (2021) found that (sexual) objectification also induces the feeling of being ostracized. Regarding the effect of ostracism on greater relative deprivation (Jiang and Chen, 2020), it is possible that individuals could feel ostracized after being objectified, and this feeling of ostracism could elicit higher relative deprivation, which in turn leads to lower prosociality. However, previous research has also indicated that ostracism could promote prosociality to some degree (e.g., Van Beest and Williams, 2011). Thus, it may help to interpret the relatively weak effect of objectification on prosociality. On that occasion, as objectified individuals feel ostracized, they may employ certain prosocial actions to regain acceptance. Given that, when exploring the effect of being objectified on prosociality in future research, the potential influence of ostracism feelings should also be taken into consideration. As the feelings of being objectified and being ostracized are intertwined with each other, greater effort should be made to distinguish their effects when investigating other impacts of the objectification experience.

Afterward, according to Study 1, objectification and prosociality were negatively correlated, which suggests a potential alternative direction of influence. That is, individuals’ prosociality could reversely influence their objectification. Prosociality was found to be associated with various psychological benefits, including happiness (Song et al., 2020), meaning in life (Klein, 2017), and even general well-being (Martela and Ryan, 2016). Recently, using experimental design, it was found that other-beneficial actions improve people’s positive affect, empathy, and social connectedness (Varma et al., 2023). As such, prosoicality could make individuals suffer less negative emotion, feel less relative deprivation, and are less self-orientated. Therefore, individuals high in prosociality would be less likely to treat others as objects. Regarding that, future research could explore whether and why prosociality could reduce individuals’ tendency to objectify others.

Moreover, even though the manipulation of objectification in the present research was effective according to manipulation check items, its effect may not be large enough to induce some of the potential consequences (i.e., less prosocial behavior). As argued by Cheng et al. (2022), the current imagining paradigm is unable to confront participants with real objectification. Previously, a collaboration game was created, in which objectified participants heard from their partners that they were selected only because they were easily manipulated people (Poon et al., 2020; Experiment 1). Additionally, Baldissarri et al. (2021) also developed a paradigm called “*The ACME Shop*,” in which participants were asked to work for a computer company with or without specific indications about the pace of their work (objectification vs. non-objectification). However, both paradigms were only applied in those scholars’ laboratory with limited samples [77 participants in the experiment by Poon et al. (2020), Experiment 1; 72 participants in the experiment by Baldissarri et al. (2021), Study 1], because those experimental paradigms would take a relatively long time to finish and come without online version. Future research may figure out how to make these paradigms more usable. For example, as suggested by Baldissarri et al. (2021), the employment of online platforms (e.g., Qualtrics) could facilitate the development of a new online paradigm.

Furthermore, although we believe that dehumanized perception of objectified individuals in the objectification process could also make them feel relatively deprived, we did not focus on that aspect. As mentioned, objectification and dehumanization are considered to be strongly related (Vaes et al., 2014), while dehumanization has been argued to be an essential aspect of objectification together with instrumentality (Baldissarri et al., 2022). Although no research has linked the objectification and dehumanization experiences directly, the objectification experience has been found to make individuals perceive themselves as less human-like (Baldissarri et al., 2021). Given that, we wondered if the dehumanization component of objectification could negatively influence individuals’ prosociality directly, rather than contribute to the relative deprivation antecedent of decreased prosociality. As self-dehumanization could increase immoral behaviors (e.g., Kouchaki et al., 2018), there might be an alternative mechanism at work by which objectification reduces individuals’ prosociality *via* their self-dehumanization. Future research could also test this model.

Additionally, although we found that objectification could also decrease individuals’ prosocial behaviors *via* relative deprivation, the main effects of objectification on prosocial behavior were not significant. That may result from two potential reasons. First, it could be the ceiling effect caused by social desirability that prevents us from detecting the main effects. Individuals tend to over-claim their prosocial behaviors when using self-report measures because of social desirability (e.g., Barry et al., 2017; Lanz et al., 2022). Future research may provide guidelines or solutions for countering or removing social desirability when assessing prosociality with self-report measures. Further research may also develop validated research paradigms for capturing prosocial behaviors, out the potential impact of social desirability. Second, there may also be a suppression effect, which means there could be another potential mediator influenced by objectification but promote prosocial behavior. For example, Dvir et al. (2021) found that being objectified could induce ostracism feeling among its victims. In that case, as prosocial individuals are more like to be liked and respected by others (e.g., Luomala et al., 2020), objectified individuals may employ more prosocial behaviors to regain more acceptance from general other people. Therefore, further research could investigate if the dropped belongingness after objectification could motivate individuals to do more prosocial behaviors.

Last, although our research replicated the negative impacts of objectification on prosociality, most of them were related only to attitudes and behavioral intentions. Future research should create and apply more reliable measurements of participants’ prosocial behaviors, especially those that could be used *via* online platforms. In Study 2, we tested participants’ prosocial behavior with a single item regarding the amount of their participation fee they would like to donate, which may carry relatively lower reliability. In recent research, Poulin et al. (2021) proposed a paradigm to measure participants’ prosocial behavior with actual actions, in which participants were invited to take part, in person, in an envelope-stuffing task for a charity project. Other scholars suggested that field experiments may more effective (e.g., Hafenbrack et al., 2020; Andreoni et al., 2021). Moreover, other indirect indicators could also be employed to capture individuals’ prosocial behaviors. For instance, Nai et al. (2018) found that individuals who tweet more prosocial concepts could be considered more prosocial, and suggested that some records of particular charity projects could also be used to indicate one’s prosociality.

## Conclusion

Our investigation focused on whether and how non-sexual objectification could impact prosociality. With both correlational design (Study 1) and experimental design (Study 2), our findings consistently support the negative relationship between objectification and prosocial intentions, which could be accounted for by relative deprivation. Although there was not enough evidence to support the effect of objectification on prosocial behavior, our findings supported the mediating effect of relative deprivation in linking these two variables in both studies. Additionally, compared to relative deprivation, negative emotions could not play the mediating role. Together, our findings suggest that objectification is an important factor in understanding interpersonal processes, while individuals’ relative deprivation could help interpret such an effect. These findings could guide future research and practices in mitigating the negative effects of objectification.

## Data availability statement

The datasets presented in this study can be found in online repositories. The names of the repository/repositories and accession number(s) can be found at: https://osf.io/dcq4h/?view_only=3f1831a5b63543e889de4280efbdeb0c.

## Ethics statement

The studies involving human participants were reviewed and approved by Human Research Ethics Committee of the University of Hong Kong. The patients/participants provided their written informed consent to participate in this study.

## Author contributions

ZZ, ZC, and K-TP contributed to conceptualization of the studies. ZZ and ZC designed the studies and contributed to the writing of the manuscript. ZZ collected and analyzed the data. TJ provided theoretical expertise and feedback in the writing process and data analysis. ZC and K-TP contributed to funding acquisition processes. All authors contributed to the article and approved the submitted version.

## Funding

This research was supported by the Hong Kong Research Grants Council’s General Research Fund (17615618), which address to ZC (Principal investigator) and K-TP (Co-Investigator).

## Conflict of interest

The authors declare that the research was conducted in the absence of any commercial or financial relationships that could be construed as a potential conflict of interest.

## Publisher’s note

All claims expressed in this article are solely those of the authors and do not necessarily represent those of their affiliated organizations, or those of the publisher, the editors and the reviewers. Any product that may be evaluated in this article, or claim that may be made by its manufacturer, is not guaranteed or endorsed by the publisher.

## References

[ref1] Adler N. E. Epel E. S. Castellazzo G. Ickovics J. R. (2000). Relationship of subjective and objective social status with psychological and physiological functioning: preliminary data in healthy, white women. Health Psychol. 19, 586–592. doi: 10.1037/0278-6133.19.6.586, PMID: 11129362

[ref2] Andreoni J. Nikiforakis N. Stoop J. (2021). Higher socioeconomic status does not predict decreased prosocial behavior in a field experiment. Nat. Commun. 12, 1–8. doi: 10.1038/s41467-021-24519-5 34253718 PMC8275767

[ref3] Andrighetto L. Baldissarri C. Gabbiadini A. Sacino A. Valtorta R. R. Volpato C. (2018). Objectified conformity: working self-objectification increases conforming behavior. Soc. Influ. 13, 78–90. doi: 10.1080/15534510.2018.1439769

[ref4] Bagozzi R. P. Moore D. J. (1994). Public service advertisements: emotions and empathy guide prosocial behavior. J. Mark. 58, 56–70. doi: 10.1177/002224299405800105

[ref5] Baldissarri C. Andrighetto L. Volpato C. (2022). The longstanding view of workers as objects: antecedents and consequences of working objectification. Eur. Rev. Soc. Psychol. 33, 81–130. doi: 10.1080/10463283.2021.1956778

[ref6] Baldissarri C. Gabbiadini A. Andrighetto L. Volpato C. (2021). The ACME shop: a paradigm to investigate working (self-) objectification. J. Soc. Psychol. 161, 526–542. doi: 10.1080/00224545.2020.1845592, PMID: 33158399

[ref7] Baldissarri C. Valtorta R. R. Andrighetto L. Volpato C. (2017). Workers as objects: the nature of working objectification and the role of perceived alienation. TPM 24, 153–166. doi: 10.4473/TPM24.2.1

[ref8] Baron S. W. (2004). General strain, street youth and crime: a test of Agnew's revised theory. Criminology 42, 457–484. doi: 10.1111/j.1745-9125.2004.tb00526.x

[ref9] Barrett L. Dunbar R. Lycett J. (2002). Human Evolutionary Psychology. Princeton, NJ: Princeton University Press.

[ref10] Barry C. T. Lui J. H. Anderson A. C. (2017). Adolescent narcissism, aggression, and prosocial behavior: the relevance of socially desirable responding. J. Pers. Assess. 99, 46–55. doi: 10.1080/00223891.2016.1193812 27362301

[ref11] Baumsteiger R. Siegel J. T. (2018). Measuring prosociality: the development of a prosocial behavioral intentions scale. J. Pers. Assess. 101, 305–314. doi: 10.1080/00223891.2017.1411918, PMID: 29448814

[ref13] Boeringer S. B. (1992). Sexual coercion among college males: assessing three theoretical models of coercive sexual behavior. Dissertation Abstracts International, 54A, 331.

[ref14] Boster F. Fediuk T. Ryan Kotowski M. (2001). The effectiveness of an altruistic appeal in the presence and absence of favors. Commun. Monogr. 68, 340–346. doi: 10.1080/03637750128074

[ref15] Breivik K. Olweus D. (2006). Children of divorce in a Scandinavian welfare state: are they less affected than US children? Scand. J. Psychol. 47, 61–74. doi: 10.1111/j.1467-9450.2006.00493.x, PMID: 16433663

[ref16] Briñol P. Petty R. E. Belding J. (2017). Objectification of people and thoughts: an attitude change perspective. Br. J. Soc. Psychol. 56, 233–249. doi: 10.1111/bjso.12183, PMID: 28188637

[ref17] Callan M. J. Kim H. Gheorghiu A. I. Matthews W. J. (2017). The interrelations between social class, personal relative deprivation, and prosociality. Soc. Psychol. Personal. Sci. 8, 660–669. doi: 10.1177/1948550616673877, PMID: 29081900 PMC5641987

[ref18] Callan M. J. Kim H. Matthews W. J. (2015). Predicting self-rated mental and physical health: the contributions of subjective socioeconomic status and personal relative deprivation. Front. Psychol. 6:1415. doi: 10.3389/fpsyg.2015.01415, PMID: 26441786 PMC4585190

[ref19] Calogero R. M. Herbozo S. Thompson J. K. (2009). Complimentary weightism: the potential costs of appearance-related commentary for women’s self-objectification. Psychol. Women Q. 33, 120–132. doi: 10.1111/j.1471-6402.2008.01479.x

[ref20] Caprara G. V. Alessandri G. Eisenberg N. (2012). Prosociality: the contribution of traits, values, and self-efficacy beliefs. J. Pers. Soc. Psychol. 102, 1289–1303. doi: 10.1037/a0025626, PMID: 21942280

[ref21] Charities Aid Foundation (2019). CAF World Giving Index. 10th Edn Charities Aid Foundation.

[ref22] Chen Z. Teng F. Zhang H. (2013). Sinful flesh: sexual objectification threatens women’s moral self. J. Exp. Soc. Psychol. 49, 1042–1048. doi: 10.1016/j.jesp.2013.07.008

[ref23] Cheng L. Li Z. Hao M. Zhu X. Wang F. (2022). Objectification limits authenticity: exploring the relations between objectification, perceived authenticity, and subjective well-being. Br. J. Soc. Psychol. 61, 622–643. doi: 10.1111/bjso.12500, PMID: 34532868

[ref24] Davis A. N. Carlo G. Schwartz S. J. Unger J. B. Zamboanga B. L. Lorenzo-Blanco E. I.. (2016). The longitudinal associations between discrimination, depressive symptoms, and prosocial behaviors in U.S. Latino/a recent immigrant adolescents. J. Youth Adolesc. 45, 457–470. doi: 10.1007/s10964-015-0394-x, PMID: 26597783 PMC11194831

[ref01] Dong Y. Zhang L. Wang H. J. Jiang J. (2023). Why is crafting the job associated with less prosocial reactions and more social undermining? The role of feelings of relative deprivation and zero-sum mindset. Journal of business ethics 184, 175–190. doi: 10.1007/s10551-022-05093-2

[ref25] Dvir M. Zhang L. Wang H. J. Jiang J. (2023). I’m up here! Sexual objectification leads to feeling ostracized. J. Pers. Soc. Psychol. 121, 332–353. doi: 10.1037/pspi0000328, PMID: 32790469

[ref26] Estrada-Hollenbeck M. Heatherton T. F. (1998). “Avoiding and alleviating guilt through prosocial behavior” in Guilt and Children. Ed. J. Bybee, (Cambridge, MA: Academic Press), 215–231.

[ref27] Falk A. Fischbacher U. (2006). A theory of reciprocity. Games Econ. Behav. 54, 293–315. doi: 10.1016/j.geb.2005.03.001

[ref28] Faul F. Erdfelder E. Lang A. G. Buchner A. (2007). G* power 3: a flexible statistical power analysis program for the social, behavioral, and biomedical sciences. Behav. Res. Methods 39, 175–191. doi: 10.3758/bf03193146, PMID: 17695343

[ref29] Fehr E. Fischbacher U. (2003). The nature of human altruism. Nature 425, 785–791. doi: 10.1038/nature02043 14574401

[ref30] Fredrickson B. L. Roberts T. A. (1997). Objectification theory. Psychol. Women Q. 21, 173–206. doi: 10.1111/j.1471-6402.1997.tb00108.x

[ref31] Fredrickson B. L. Roberts T. A. Noll S. M. Quinn D. M. Twenge J. M. (1998). That swimsuit becomes you: sex differences in self-objectification, restrained eating, and math performance. J. Pers. Soc. Psychol. 75, 269–284. doi: 10.1037/0022-3514.75.1.269 9686464

[ref32] Galen L. W. (2012). Does religious belief promote prosociality? A critical examination. Psychol. Bull. 138, 876–906. doi: 10.1037/a0028251, PMID: 22925142

[ref33] Gruenfeld D. H. Inesi M. E. Magee J. C. Galinsky A. D. (2008). Power and the objectification of social targets. J. Pers. Soc. Psychol. 95, 111–127. doi: 10.1037/0022-3514.95.1.111, PMID: 18605855

[ref34] Hafenbrack A. C. Cameron L. D. Spreitzer G. M. Zhang C. Noval L. J. Shaffakat S. (2020). Helping people by being in the present: mindfulness increases prosocial behavior. Organ. Behav. Hum. Decis. Process. 159, 21–38. doi: 10.1016/j.obhdp.2019.08.005

[ref36] Hayes A. F. (2012). PROCESS: a versatile computational tool for observed variable mediation, moderation, and conditional process modeling [white paper]. Available at: www.afhayes.com/public/process2012.pdf

[ref37] Holland E. Koval P. Stratemeyer M. Thomson F. Haslam N. (2017). Sexual objectification in women's daily lives: a smartphone ecological momentary assessment study. Br. J. Soc. Psychol. 56, 314–333. doi: 10.1111/bjso.12152, PMID: 27484394

[ref38] Jiang T. Chen Z. (2020). Relative deprivation: a mechanism for the ostracism–aggression link. Eur. J. Soc. Psychol. 50, 347–359. doi: 10.1002/ejsp.2621

[ref39] Jiang T. Sedikides C. (2021). Awe motivates authentic-self pursuit via self-transcendence: implications for prosociality. J. Pers. Soc. Psychol. 123, 576–596. doi: 10.1037/pspi0000381, PMID: 34855435

[ref40] Klein N. (2017). Prosocial behavior increases perceptions of meaning in life. J. Posit. Psychol. 12, 354–361. doi: 10.1080/17439760.2016.1209541

[ref41] Kouchaki M. Dobson K. S. H. Waytz A. Kteily N. S. (2018). The link between self-dehumanization and immoral behavior. Psychol. Sci. 29, 1234–1246. doi: 10.1177/0956797618760784, PMID: 29787345

[ref42] Lanz L. Thielmann I. Gerpott F. H. (2022). Are social desirability scales desirable? A meta-analytic test of the validity of social desirability scales in the context of prosocial behavior. J. Pers. 90, 203–221. doi: 10.1111/jopy.12662, PMID: 34265863

[ref43] Luomala H. Puska P. Lähdesmäki M. Siltaoja M. Kurki S. (2020). Get some respect–buy organic foods! When everyday consumer choices serve as prosocial status signaling. Appetite 145:104492. doi: 10.1016/j.appet.2019.104492, PMID: 31654656

[ref44] Malti T. Gasser L. Buchmann M. (2009). Aggressive and prosocial children's emotion attributions and moral reasoning. Aggress. Behav. 35, 90–102. doi: 10.1002/ab.20289, PMID: 18985747

[ref45] Martela F. Ryan R. M. (2016). Prosocial behavior increases well-being and vitality even without contact with the beneficiary: causal and behavioral evidence. Motiv. Emot. 40, 351–357. doi: 10.1007/s11031-016-9552-z

[ref46] Moradi B. Huang Y. P. (2008). Objectification theory and psychology of women: a decade of advances and future directions. Psychol. Women Q. 32, 377–398. doi: 10.1111/j.1471-6402.2008.00452.x

[ref47] Nai J. Narayanan J. Hernandez I. Savani K. (2018). People in more racially diverse neighborhoods are more prosocial. J. Pers. Soc. Psychol. 114, 497–515. doi: 10.1037/pspa0000103, PMID: 29620398

[ref48] Nussbaum M. C. (1995). Objectification. Philos Public Aff 24, 249–291. doi: 10.1111/j.1088-4963.1995.tb00032.x

[ref49] Penner L. A. Dovidio J. F. Piliavin J. A. Schroeder D. A. (2005). Prosocial behavior: multilevel perspectives. Annu. Rev. Psychol. 56, 365–392. doi: 10.1146/annurev.psych.56.091103.070141, PMID: 15709940

[ref50] Penner L. A. Midili A. R. Kegelmeyer J. (1997). Beyond job attitudes: a personality and social psychology perspective on the causes of organizational citizenship behavior. Human Performance 10, 111–131. doi: 10.1207/s15327043hup1002_4

[ref51] Perugini M. Gallucci M. Presaghi F. Ercolani A. P. (2003). The personal norm of reciprocity. Eur. J. Personal. 17, 251–283. doi: 10.1002/per.474

[ref53] Poon K. T. Chen Z. Teng F. Wong W. Y. (2020). The effect of objectification on aggression. J. Exp. Soc. Psychol. 87:103940. doi: 10.1016/j.jesp.2019.103940

[ref54] Poulin M. J. Ministero L. M. Gabriel S. Morrison C. D. Naidu E. (2021). Minding your own business? Mindfulness decreases prosocial behavior for people with independent self-Construals. Psychol. Sci. 32, 1699–1708. doi: 10.1177/09567976211015184, PMID: 34705576

[ref55] Prentice M. Jayawickreme E. Hawkins A. Hartley A. Furr R. M. Fleeson W. (2019). Morality as a basic psychological need. Soc. Psychol. Personal. Sci. 10, 449–460. doi: 10.1177/1948550618772011

[ref56] Quinn D. M. Kallen R. W. Twenge J. M. Fredrickson B. L. (2006). The disruptive effect of self-objectification on performance. Psychol. Women Q. 30, 59–64. doi: 10.1111/j.1471-6402.2006.00262.x

[ref57] Rand D. G. (2012). The promise of mechanical Turk: how online labor markets can help theorists run behavioral experiments. J. Theor. Biol. 299, 172–179. doi: 10.1016/j.jtbi.2011.03.004, PMID: 21402081

[ref58] Raskauskas J. L. Gregory J. Harvey S. T. Rifshana F. Evans I. M. (2010). Bullying among primary school children in New Zealand: relationships with prosocial behaviour and classroom climate. Educ. Res. 52, 1–13. doi: 10.1080/00131881003588097

[ref59] Roberts T. A. Gettman J. Y. (2004). Mere exposure: gender differences in the negative effects of priming a state of self-objectification. Sex Roles 51, 17–27. doi: 10.1023/B:SERS.0000032306.20462.22

[ref60] Shedd J. R. (2008). When peace agreements create spoilers: the Russo-Chechen agreement of 1996. Civil Wars 10, 93–105. doi: 10.1080/13698240802062648

[ref61] Shi J. Wang X. Teng F. Chen Z. (2022). A little appreciation goes a long way: gratitude reduces objectification. J. Posit. Psychol., 18, 627–635. doi: 10.1080/17439760.2022.2053877

[ref62] Simpson B. Willer R. (2015). Beyond altruism: sociological foundations of cooperation and prosocial behavior. Annu. Rev. Sociol. 41, 43–63. doi: 10.1146/annurev-soc-073014-112242

[ref63] Smith H. J. Pettigrew T. F. (2015). Advances in relative deprivation theory and research. Soc. Justice Res 28, 1–6. doi: 10.1007/s11211-014-0231-5

[ref64] Smith H. J. Pettigrew T. F. Pippin G. M. Bialosiewicz S. (2012). Relative deprivation: a theoretical and meta-analytic review. Personal. Soc. Psychol. Rev. 16, 203–232. doi: 10.1037/pspi0000058 22194251

[ref65] Sober E. Wilson D. S. (1999). Unto Others: The Evolution and Psychology of Unselfish Behavior. Cambridge, MA: Harvard University Press.

[ref66] Song Y. Broekhuizen M. L. Dubas J. S. (2020). Happy little benefactor: prosocial behaviors promote happiness in young children from two cultures. Front. Psychol. 11:1398. doi: 10.3389/fpsyg.2020.01398, PMID: 32714246 PMC7346734

[ref67] Teng F. Chen Z. Poon K. T. Zhang D. (2015). Sexual objectification pushes women away: the role of decreased likability. Eur. J. Soc. Psychol. 45, 77–87. doi: 10.1002/ejsp.2070

[ref68] Twenge J. M. Baumeister R. F. DeWall C. N. Ciarocco N. J. Bartels J. M. (2007). Social exclusion decreases prosocial behavior. J. Pers. Soc. Psychol. 92, 56–66. doi: 10.1037/0022-3514.92.1.56 17201542

[ref69] Vaes J. Loughnan S. Puvia E. (2014). “The inhuman body: when sexual objectification become dehumanizing” in Humanness and Dehumanization. eds. Bain P. Vaes J. Leyens J.-P. (New York: Taylor & Francis), 186–204.

[ref70] Van Beest I. Williams K. D. (2011). “Why hast thou forsaken me?” the effect of thinking about being ostracized by god on well-being and prosocial behavior. Soc. Psychol. Personal. Sci., 2, 379–386. doi: 10.1177/1948550610393312

[ref71] Varma M. M. Chen D. Lin X. Aknin L. B. Hu X. (2023). Prosocial behavior promotes positive emotion during the COVID-19 pandemic. Emotion 23, 538–553. doi: 10.1037/emo0001077, PMID: 35298223

[ref72] Wedekind C. Braithwaite V. A. (2002). The long-term benefits of human generosity in indirect reciprocity. Curr. Biol. 12, 1012–1015. doi: 10.1016/S0960-9822(02)00890-4 12123575

[ref73] Xiong M. Xiao L. Ye Y. (2021). Relative deprivation and prosocial tendencies in Chinese migrant children: testing an integrated model of perceived social support and group identity. Front. Psychol. 12:2161. doi: 10.3389/fpsyg.2021.658007, PMID: 34168590 PMC8217643

[ref74] Zhang H. Liu M. Tian Y. (2016). Individual-based relative deprivation (IRD) decreases prosocial behaviors. Motiv. Emot. 40, 655–666. doi: 10.1007/s11031-016-9564-8

